# Imatinib Mesylate Reduces Voiding Frequency in Female Mice With Acute Cyclophosphamide-Induced Cystitis

**DOI:** 10.3389/fnsys.2022.867875

**Published:** 2022-05-13

**Authors:** Megan E. Perkins, Beatrice M. Girard, Susan E. Campbell, Margaret A. Vizzard

**Affiliations:** Department of Neurological Sciences, Larner College of Medicine, University of Vermont, Burlington, VT, United States

**Keywords:** interstitial cystitis, interstitial cells, platelet-derived growth factor receptor (PDGFR), imatinib mesylate, conscious cystometry, somatic sensitivity

## Abstract

Lamina propria interstitial cells that express the tyrosine kinase receptor, platelet-derived growth factor receptor alpha (PDGFRα) may play a role in urinary sensory signaling. Imatinib mesylate, also referred to as imatinib, is a tyrosine kinase inhibitor that can inhibit PDGFRα and has been widely used in urological research. We evaluated the functional effects of imatinib administration (via oral gavage or intravesical infusion) with two different experimental designs (prevention and treatment), in a cyclophosphamide (CYP)-induced cystitis (acute, intermediate, and chronic), male and female rodent model using conscious cystometry and somatic sensitivity testing. Imatinib significantly (0.0001 ≤ *p* ≤ 0.05) decreased voiding frequency and increased bladder capacity in acute CYP-induced cystitis, by the prevention (females) and treatment (females and males) designs. Imatinib was not effective in preventing or treating intermediate or chronic CYP-induced cystitis in either sex. Interestingly, in the prevention experiments, imatinib administration increased (0.0001 ≤ *p* ≤ 0.01) voiding frequency and decreased bladder capacity in control mice. However, in the treatment experiments, imatinib administration decreased (0.01 ≤ *p* ≤ 0.05) voiding frequency and increased bladder capacity in control mice. Bladder function improvements observed with imatinib treatment in acute CYP-induced cystitis mice remained and additionally improved with a second dose of imatinib 24 hours after CYP treatment. Imatinib administration did not affect pelvic somatic sensitivity in female mice with acute CYP-induced cystitis. Our studies suggest that (1) imatinib improves bladder function in mice with acute CYP-induced cystitis with a prevention and treatment design and (2) interstitial cells may be a useful target to improve bladder function in cystitis.

## Introduction

The storage and release functions of the urinary bladder are highly organized and coordinated by peripheral and central neural pathways involving the spinal cord, brain, afferent and efferent nerves, dorsal root ganglia (DRG), autonomic ganglia and the urinary bladder (Andersson, 2002; de Groat et al., 2015). The urinary bladder is organized into layers: the mucosa, muscularis propria, and the adventitia/serosa. The mucosal layer consists of transitional epithelial (urothelial) cells that line the lumen of the bladder and a lamina propria (LP) (also referred to as the submucosa) beneath the basement membrane of the epithelial cells (Andersson, 2002, 2004; Merrill et al., 2016). The urothelium responds to stimuli and releases various factors including ATP, ACh, and nitric oxide (Birder and Andersson, 2013; Merrill et al., 2016). The urothelium can be compromised with injury or inflammation, allowing toxic substances to reach the suburothelial nerve plexus and muscular layers, contributing to urinary urgency, frequency, and pain during voiding (Birder, 2005; Birder and Andersson, 2013). The LP is composed of loose connective tissue, interstitial cells, vasculature, adipocytes, lymphatic vessels, fibroblasts, nerve fibers and endings and may integrate urothelial and smooth muscle input (Andersson, 2002, 2004; Birder and Andersson, 2013).

Neuro-urological research continues to advance our understanding of the mechanisms underlying bladder function and dysfunction. However, many questions remain concerning the identity of potential treatment targets as well as treatment options for urinary bladder dysfunction. For example, current theories suggest a sensory intermediary function for the LP interstitial cells to mediate signals from the urothelium to afferent nerves and/or the detrusor muscle, to regulate bladder sensation and contractility (Gray et al., 2013; Heppner et al., 2017; Koh et al., 2018). LP interstitial cells in the urinary bladder are positioned near afferent nerve terminals in the suburothelial plexus (Koh et al., 2012; Gabella, 2019) and exhibit cholinergic (Mukerji et al., 2006; Grol et al., 2009), purinergic (Sui et al., 2006; Fry et al., 2012; Lee et al., 2014; Heppner et al., 2017) and nitrergic (Smet et al., 1996; Gillespie et al., 2006; de Jongh et al., 2007) properties (Koh et al., 2018). Other potential roles for LP interstitial cells include: transduction of bladder mechanosensation (Isogai et al., 2016; Heppner et al., 2017; Liu et al., 2018; Steiner et al., 2018; Dalghi et al., 2019), propagation of calcium transients (Heppner et al., 2017) and signal transmission via gap junctions (Ikeda et al., 2007). A subset of LP interstitial cells expresses the tyrosine kinase receptor platelet-derived growth factor receptor alpha (PDGFRα) (Koh et al., 2012; Monaghan et al., 2012; Sancho et al., 2017) and this subpopulation may be important for bladder voiding during early postnatal development before coordinated urinary function (Kanai et al., 2007; Heppner et al., 2017). PDGFRα+ interstitial cells are found in the urinary bladder of human, mouse, rat, guinea pig, and pig, with similar morphology, chemical expression, and organization (Gevaert et al., 2014b,^2017^; Steiner et al., 2018). Studies suggest that PDGFRα+ LP interstitial cells may also contribute to bladder dysfunction (e.g., interstitial cystitis/bladder pain syndrome, IC/BPS) because preclinical rodent models show increased expression of interstitial cell markers (de Jongh et al., 2007; Kubota et al., 2008; Kim et al., 2011; Abrams et al., 2012; Fry et al., 2012; Johnston et al., 2012; Deng et al., 2015; Kjell et al., 2015; Liu et al., 2017, 2018; Sancho et al., 2017). PDGFRα+ bladder interstitial cells also exhibit morphological and chemical differences in patients with OAB or IC/BPS (Neuhaus et al., 2005; Roosen et al., 2009a,b; Gevaert et al., 2011, 2015; Meng et al., 2015). Additionally, interstitial cells display abnormal electrophysiological properties in IC/BPS and may contribute to the increased and spontaneous contractions observed in the detrusor (Rosenberg et al., 2016; Liu et al., 2017, 2018).

Imatinib mesylate, also referred to as imatinib, is a competitive inhibitor of the ATP-binding site of certain receptor tyrosine kinases including PDGFRα (Buchdunger et al., 1996, 2000; Kilic et al., 2000) and has been commonly used in lower urinary tract (LUT) studies to target PDGFRα+ interstitial cells (Biers et al., 2006; Kubota et al., 2006; Min et al., 2011; Abrams et al., 2012; Deng et al., 2013; Gevaert et al., 2014a; Kjell et al., 2015; Sancho et al., 2017; Giglio et al., 2018). Recent reports indicate that imatinib administration reduces PDGFRα expression and voiding frequency in rodents with IC/BPS (Sancho et al., 2017; Giglio et al., 2018). In this study, we expand upon previous studies (Sancho et al., 2017; Giglio et al., 2018) to determine the functional effects of imatinib mesylate administration (e.g., gavage, intravesical) in a cyclophosphamide (CYP)-induced cystitis (acute, intermediate, chronic) mouse model (male and female) using conscious, unrestrained, open-outlet cystometry. We also assess the mechanical sensitivity of the pelvic region in a CYP-induced cystitis mouse model with and without imatinib treatment (e.g., gavage, intravesical).

## Materials and Methods

### Experimental Animals

Male and female wildtype (WT), C57BL/6 mice (3–5 months) (Jackson Labs, Bar Harbor, ME, United States) were used in these studies. Mice were bred locally at The Larner College of Medicine, University of Vermont (UVM) animal facilities in standard laboratory conditions, as previously described (Tooke et al., 2019). The UVM Institutional Animal Care and Use Committee (IACUC) approved all experimental procedures involving animal use (IACUC #X9-020). The UVM Office of Animal Care and Management managed animal care in accordance with the American Association for Accreditation of Laboratory Animal Care (AAALAC) and National Institutes of Health (NIH) guidelines. All efforts were taken to minimize animal pain and distress. Mice exhibiting signs of pain and distress that could not be managed with post-operative analgesics were immediately euthanized. Separate cohorts of littermate WT mice were used in these studies. Female mice were primarily used because of the increased IC/BPS prevalence in human females (Hanno et al., 2015). The estrous cycle status of mice was not determined.

### Cyclophosphamide Dosing and Administration

Cyclophosphamide treatment groups received CYP injections (i.p.) to induce acute (200 mg/kg, 4 h), intermediate (200 mg/kg, 48 h) or chronic (75 mg/kg, every 72 h for 8 days) urinary bladder inflammation as previously described (Malley and Vizzard, 2002; Bjorling et al., 2011; Guo et al., 2018). Control groups did not receive CYP. After completion of CYP treatment, mice were used for bladder function testing (i.e., cystometry) or tissues (e.g., urinary bladder) were harvested from anesthetized (5% isoflurane in oxygen) mice subsequently euthanized by thoracotomy. Urinary bladders were collected and stored for future experiments.

### Imatinib Mesylate Dosing and Administration

The dose and route of imatinib were determined in pilot studies and by using previous reports (Abrams et al., 2012; Kjell et al., 2015; Sancho et al., 2017) as guidance. Imatinib or vehicle (i.e., water or saline) was delivered by two different routes in separate cohorts of mice: (1) systemically by oral gavage (250 mg/kg; 22 gauge/25 mm, stainless steel); or (2) directly by intravesical infusion (50 μM) via an externalized bladder catheter, or by transurethral intravesical infusion (50 μM). Upon cessation of gavage and infusion, mice were used for testing and/or euthanized for tissue collection.

#### Oral Gavage (Prevention Schedule)

Mice were placed headfirst in a cone shaped, plastic bag with the far corner cut. Alternative handling techniques (i.e., scruff) could not be used because of the intravesical implant surgery with exteriorization at the base of the neck. Mice were monitored constantly to ensure correct gavage needle placement and animal well-being. The same individual performed all gavage procedures at the same time of day (8 a.m.–12 p.m.) to reduce potential variability.

Mice were pre-treated with imatinib (250 mg/kg) for 5 days prior to CYP (i.p.) administration ranging from 5 to 12 days total, depending on the CYP dosing schedule. All groups were gavaged daily throughout the entire experimental period with imatinib or vehicle control, including on surgery, CYP administration and cystometry testing days. On these days, mice were gavaged and left undisturbed in their home cages for at least 30 min prior to surgery, CYP administration or cystometry testing.

#### Catheter Implant for Intravesical Infusion (Prevention Schedule)

Mice were pre-treated with imatinib (50 μM, 30 min, 0.5 mL, 1X/day) via an externalized bladder catheter for 5 days prior to CYP (4 h, 200 mg/kg, i.p.) administration. Some cohorts additionally received protamine sulfate (PS; 10 mg/kg, 0.5 mL, 30 min) infusions, prior to imatinib. Bladder parameters were assessed using conscious, open outlet cystometry after acute (4 h) CYP.

#### Catheter Implant for Intravesical Infusion (Treatment Schedule)

Acute (4 h, 200 mg/kg, i.p.) or chronic (every 72 h for 8 days, 75 mg/kg, i.p.) cystitis was induced with CYP as previously described (Guo et al., 2018; Tooke et al., 2019). Bladder parameters were assessed using conscious, open outlet cystometry before and after the imatinib infusion (50 μM, 30 min, 0.5 mL).

#### Transurethral Intravesical Infusion (Treatment Schedule)

Mice were anesthetized with isoflurane in oxygen (3–4%), placed in the supine position on a water mat heating pad and catheterized by inserting lubricated polyethylene tubing (PE-10, Clay Adams, Parsippany, NJ, United States) into the bladder through the urethra. The mice were manually voided before the urethral instillation procedure. Solution (i.e., imatinib or saline) was instilled into the bladder until a full bladder could be palpated (25–400 μL). The catheter was then removed from the urethra while the mice remained anesthetized (30 min).

### Intravesical Catheter Implantation

Intravesical catheter implantation was performed as previously described (Schnegelsberg et al., 2010; Gonzalez et al., 2013). Mice received analgesics (carprofen, 0.1 mg/kg, s.c.) before and after (every 24 h for 48 h total) surgery.

### Conscious, Open-Outlet Cystometry With Continuous Infusion of Saline

Conscious, open-outlet cystometry with continuous saline infusion was performed as previously described (Guo et al., 2018; Tooke et al., 2019). Three days after surgery, mice were placed, unrestrained, in a wire bottom cage with the dorsal neck tubing exteriorized and connected to the cystometry system (Med Associates, St. Albans, VT, United States). Room temperature saline was infused into the bladder (25 μl/min) to elicit repetitive bladder contractions. After an initial accommodation period (20–30 min), a minimum of 6 consistent micturition cycles were recorded. Urodynamic measures included: minimum pressure (pressure at the beginning of bladder filling), threshold pressure (bladder pressure immediately before micturition), maximum pressure, intermicturition interval (time between micturition events) and infused volume. Bladder capacity is defined as the infused volume necessary to elicit a micturition event. Voided volume was not analyzed as multiple groups (e.g., CYP groups) were unable to be assessed, due to very small volume voids. Non-voiding contractions (NVCs) were also not analyzed because multiple groups did not reliably produce NVCs.

Imatinib (50 μM) was intravesically infused into the bladder for some experiments where mice served as their own controls and were evaluated before and after imatinib treatment. Baseline bladder function testing was conducted with intravesical saline, followed by intravesical imatinib treatment, then bladder function testing was repeated. At least 6, reproducible micturition cycles were obtained before and after imatinib treatment. After cystometry, mice were euthanized by thoracotomy. Urinary bladders were harvested for future experiments.

### Pelvic Mechanical Sensitivity Testing

Mechanical sensitivity was assessed in separate groups of mice that did not undergo the intravesical catheter implantation surgery nor cystometry. The imatinib prevention (gavage) and treatment schedules (intravesical infusion) were evaluated. All mice were acclimated to the testing environment for 2 h/day for 2 days prior to Schnegelsberg et al. (2010) and on the test day.

#### Prevention

Cohorts received daily doses of imatinib or vehicle (water) via oral gavage, 1X/day for 5 days. On the test day (i.e., last day), the cohorts received the final dose of imatinib, followed 30 min later with acute CYP treatment. Mechanical sensitivity was evaluated 4 h after CYP treatment.

#### Treatment

Cohorts received acute CYP treatment followed 4 h later by an intravesical infusion (transurethral catheter) of imatinib or vehicle (saline) followed (30 min) by mechanical sensitivity testing.

Control mice did not receive CYP treatment. Mice were tested in individual plexiglass chambers with a stainless-steel wire grid floor, using calibrated von Frey microfilaments (0.008–1 g, Semmes Weinstein, Stoelting Co, Wood Dale, IL, United States). Microfilaments were applied in a perpendicular manner, until the hair buckled to the lower pelvic/abdominal region overlying the bladder for 1–3 s with an interstimulus interval of at least 15 s. Positive behaviors included sharp retraction of the abdomen, jumping or immediate licking or scratching of the affected area (May and Vizzard, 2010; Schnegelsberg et al., 2010). Mechanical sensitivity testing was performed in a blinded manner, with mixed treatment and control groups. The groups were decoded following analyses.

### Mechanical Sensitivity Analysis

The mechanical force needed to elicit a withdrawal response in 50% of the mice was determined using the up-down method (Deuis et al., 2017). At least four responses were obtained after the first change in von Frey filament direction. The withdrawal threshold was defined as a positive response, followed by a negative response in either direction, repeated at least twice. Two separate evaluators determined the withdrawal thresholds; one evaluator was blinded to all conditions.

### Pain Behavioral Assessments

Secondary behavioral assessments that may be indicative of pain or distress were also conducted. Behavior was only assessed in the control and treatment (intravesical) groups also undergoing mechanical sensitivity testing. Mice were acclimated to individual cages 30 min/day, for 2 days before behavioral assessments. On the test day, after microfilament sensitivity testing, mice were immediately placed in individual cages for acclimation (10 min). Mice were then recorded using a video camera for 15 min. An evaluator, blinded to groups, examined the video and determined total time moving and total licking behaviors of the abdominal pelvic area (Boucher et al., 2000; Arras et al., 2007; Yoshikawa et al., 2012).

### Exclusionary Criteria

Mice were withdrawn from study because of adverse postoperative events or pain, lethargy, or if distress could not be alleviated by postoperative analgesics. Some cystometry recordings were rendered unusable due excessive behavioral movements (e.g., grooming, standing, walking, defecation, and chewed tubing). Approximately 10% of mice (n = 32) were removed from the study.

### Statistical Analyses

All values represent mean ± SEM. When the *F* ratio exceeded the critical value at α = 0.05, *post hoc* multiple comparisons tests were performed. Data were analyzed using a two-way analysis of variance (ANOVA) with Bonferroni’s multiple comparisons test, a repeated measures two-way ANOVA with Šídák’s multiple comparisons test, or Student’s paired *t*-test, as indicated by experimental design. All analyses were performed using GraphPad Prism software (GraphPad Prism version 8.0.0 for Windows, GraphPad Software, San Diego, CA, United States).^1^

## Results

### Imatinib Decreases Voiding Frequency and Increases Bladder Capacity in the Acute (4 h) Cyclophosphamide-Induced Cystitis Model: Prevention or Treatment Design

**FIGURE 1:**
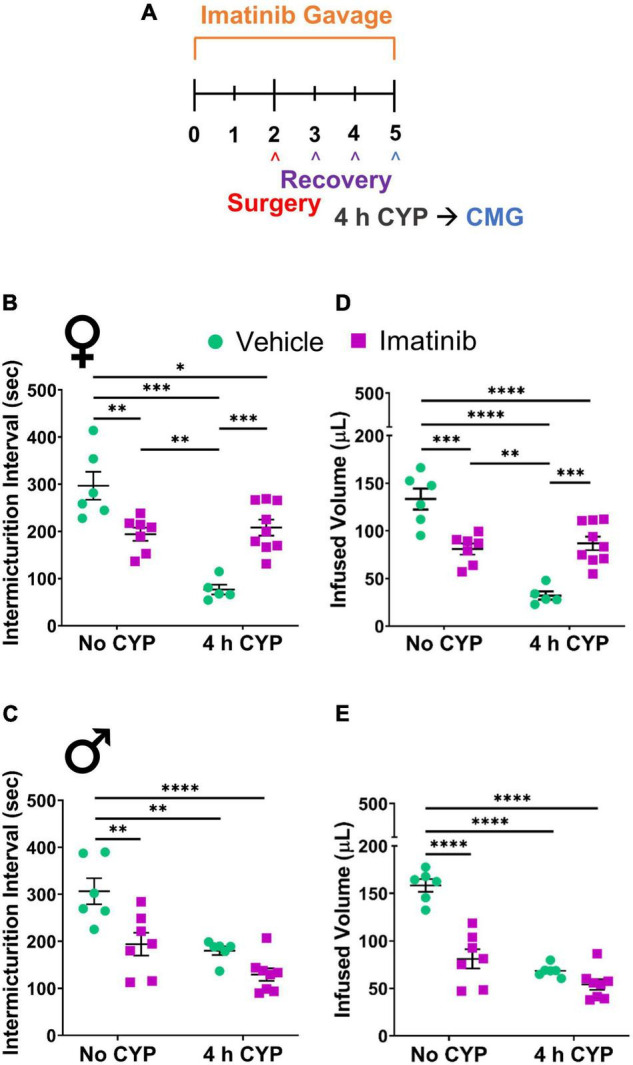
Imatinib mesylate pre-treatment via oral gavage significantly alters intermicturition interval (IMI) and infused volume (IV) in an acute (4 h) cyclophosphamide (CYP)-induced cystitis mouse model. **(A)** Acute CYP-induced cystitis (200 mg/kg, 4 h, i.p.) experimental schedule for imatinib mesylate pre-treatment via oral gavage (250 mg/kg, 5 days, 1X/day). **(B,D)** Imatinib mesylate pre-treatment significantly increased IMI and IV (*p* ≤ 0.001) in female mice with acute (4 h) CYP-induced cystitis. **(C,E)** Imatinib mesylate pre-treatment did not significantly affect bladder measures in male mice with acute CYP-induced cystitis. **(B–E)** Imatinib pre-treatment alone by gavage, without CYP, significantly (0.01 ≤ *p* ≤ 0.0001) decreased IMI and IV in both female and male mice, compared to vehicle treatment alone. *n* = 5–9. Values are mean ± SEM. *p ≤ 0.05; ^**^p ≤ 0.01; ^***^p ≤ 0.001; ^****^p ≤ 0.0001 by two-way ANOVA with Bonferroni’s multiple comparisons test. CMG, cystometrogram.

**FIGURE 2:**
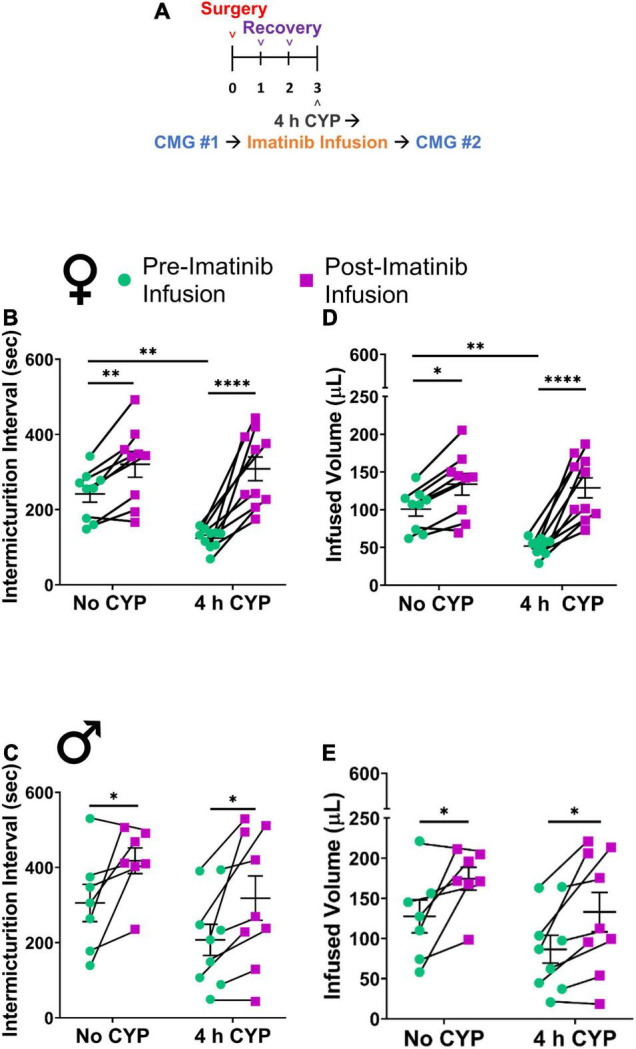
Imatinib mesylate treatment via intravesical infusion significantly increases IMI and IV in an acute (4 h) CYP-induced cystitis mouse model. **(A)** Acute CYP-induced cystitis (200 mg/kg, 4 h, i.p.) experimental schedule for imatinib mesylate treatment via intravesical infusion (50 μM, 30 min, 0.5 mL). **(B–E)** Imatinib treatment via intravesical infusion significantly increases the IMI and IV in female (*p* ≤ 0.0001) and male (*p* ≤ 0.05) with acute (4 h) CYP-induced cystitis. Imatinib treatment via intravesical infusion significantly increases the IMI and IV in female (*p* ≤ 0.01) and male (*p* ≤ 0.05) control (no CYP) mice. Control (no CYP) and 4 h CYP treated mice were separate groups of mice, each tested before and after imatinib infusion. *n* = 6–10. Values are mean ± SEM. *p ≤ 0.05; ^**^p ≤ 0.01; ^****^p ≤ 0.0001 by two-way repeated measures ANOVA with Šídák’s multiple comparisons test. CMG, cystometrogram.

Administration of imatinib significantly (*p* ≤ 0.001) increased the intermicturition interval (IMI) and the infused volume (IV) in mice with acute (200 mg/kg, i.p., 4 h) CYP-induced cystitis when evaluated in two different drug delivery methods and experimental designs (Figures 1, 2).

#### Prevention Effects

**FIGURE 3:**
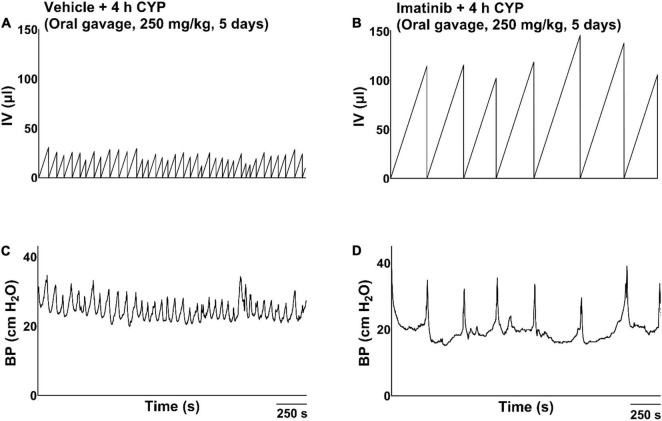
Representative bladder function cystometry recordings from female mice with acute CYP-induced cystitis, pre-treated with imatinib mesylate or vehicle via oral gavage. **(A,B)** Mice with 4 h CYP-induced cystitis (200 mg/kg, 4 h, i.p.) pre-treated with vehicle exhibited significantly decreased IMI (*p* ≤ 0.001) and IV (*p* ≤ 0.0001), compared to control mice. **(C,D)** Mice with 4 h CYP-induced cystitis pre-treated with imatinib (250 mg/kg, 5 days, 1X/day) exhibited significantly (*p* ≤ 0.001) increased IMI and IV, compared to mice with 4 h CYP-induced cystitis pre-treated with vehicle. Recordings are from separate cohorts of mice. *n* = 5–9. By two-way ANOVA with Bonferroni’s multiple comparisons test. CMG, cystometrogram; BP, bladder pressure.

Mice were pre-treated daily via oral gavage or direct intravesical infusion with imatinib mesylate, followed by induction of CYP-induced cystitis (Figure 1A). Female and male mice with acute (4 h) CYP-induced cystitis exhibited significantly (*p* ≤ 0.01) decreased IMI and IV compared to controls, as expected (Figures 1B–E, 3A,B). In female mice with acute (4 h) CYP-induced cystitis, imatinib (250 mg/kg, 5 days) administration via oral gavage significantly (*p* ≤ 0.001) increased IMI and IV compared to vehicle (water) treated mice (Figures 1B,D, 3C,D). However, this imatinib delivery method and treatment schedule did not affect IMI or IV in male mice with CYP-induced cystitis (Figures 1C,E). Imatinib pre-treatment significantly (*p* ≤ 0.01) increased bladder pressures (minimum, threshold, maximum) in male and female mice with acute CYP-induced cystitis, compared to controls (Table 1).

**TABLE 1:** Mean bladder pressures during conscious, open-outlet cystometry in control (no CYP), acute CYP (4 h, 200 mg/kg, i.p.), or chronic CYP (75 mg/kg every 72 h for 8 days, i.p.) male and female mice pre-treated with imatinib mesylate or vehicle via oral gavage.

Group	Threshold pressure	Minimum pressure	Maximum pressure
*Female mice*			
Vehicle (No CYP)	12.09 ± 0.7	10.23 ± 0.7	27.04 ± 2.1
Vehicle + Acute (4 h) CYP	28.84 ± 2.9**	24.12 ± 2.9**	34.25 ± 3.0
Imatinib (No CYP)	27.35 ± 2.1**	18.9 ± 1.4	38.82 ± 4.0
Imatinib + Acute (4 h) CYP	39.34 ± 3.7^+^****	32.41 ± 3.0^++^****	50.83 ± 4.8**
Vehicle (No CYP)	21.71 ± 2.9	13.77 ± 1.1	34.06 ± 3.5
Vehicle + Chronic (8 d) CYP	33.5 ± 2.7^+^*	27.88 ± 2.8^+^**	44.87 ± 2.7
Imatinib (No CYP)	22.22 ± 1.5	16.45 ± 1.5	34.68 ± 2.0
Imatinib + Chronic (8 d) CYP	30.45 ± 2.7	26.04 ± 3.1**	42.77 ± 3.6
*Male Mice*			
Vehicle (No CYP)	12.65 ± 0.5	11.82 ± 0.2	28.71 ± 1.3
Vehicle + Acute (4 h) CYP	17.26 ± 0.4^++^	15.53 ± 0.8	34.22 ± 1.4
Imatinib (No CYP)	26.23 ± 2.1****	20.58 ± 2.0**	38.86 ± 2.9*
Imatinib + Acute (4 h) CYP	24.22 ± 1.7^#^***	21.13 ± 1.6**	35.37 ± 2.1

*n = 5–9. Values are means ± SEM by two-way ANOVA with Bonferroni’s multiple comparisons test.*

*p ≤ 0.05; **p ≤ 0.01; ***p ≤ 0.001; ****p ≤ 0.0001 when compared to vehicle control, ^+^p ≤ 0.05; ^++^p ≤ 0.01; ^++++^p ≤ 0.0001 when compared to imatinib control, or ^#^p ≤ 0.05; ^##^p ≤ 0.01; ^####^p ≤ 0.0001 when compared to vehicle + acute CYP.

The systemic administration of imatinib by gavage may have off-target effects not specifically due to PDGFRα inhibition at the level of the urinary bladder. Thus, we conducted a series of experiments to intravesically infuse imatinib to investigate bladder-specific effects of PDGFRα inhibition. Mice were pre-treated with imatinib (50 μM, 0.5 mL, 30 min) for 5 days and acute cystitis was induced via CYP on day 5. This treatment and dosing schedule resulted in mice exhibiting increased voiding frequency as documented in mice with only CYP-induced cystitis (4 h) (data not shown). We hypothesized that intravesical imatinib was unable to penetrate the intact urothelial barrier to access the LP. Thus, we performed a follow-up study in a separate cohort of mice that received intravesical protamine sulfate (PS) (10 mg/kg, 0.5 mL, 30 min) every other day, prior to imatinib infusions, to disrupt the urothelial lining (Stein et al., 1996). PS does not affect voiding frequency at this concentration (Chuang et al., 2003; Fraser et al., 2003; Klinger and Vizzard, 2008). Urinary bladder function resembled that in CYP-treated mice (without imatinib) but imatinib treatment did not significantly affect IMI or IV, despite PS treatment (data not shown).

#### Treatment Effects

**FIGURE 4:**
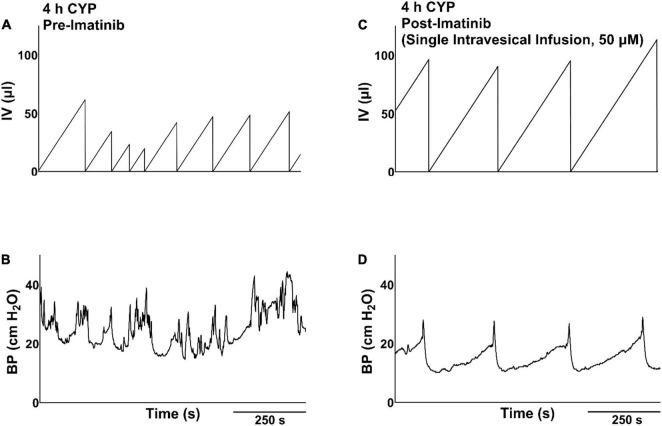
Representative bladder function cystometry recordings from a female mouse with acute CYP-induced cystitis, before and after imatinib mesylate treatment via intravesical infusion. **(A,B)** Before imatinib treatment via intravesical infusion, mice with acute CYP-induced cystitis (200 mg/kg, 4 h, i.p.) exhibited decreased IMI and IV. **(C,D)** After imatinib treatment via intravesical infusion (50 μM, 30 min, 0.5 ml), mice with 4 h CYP-induced cystitis exhibited significantly (*p* ≤ 0.0001) increased IMI and IV. Recordings displayed are from the same mouse, before and after imatinib treatment. Mice with 4 h CYP-induced cystitis in these experiments acted as their own controls. *n* = 6–10. By two-way repeated measures ANOVA with Šídák’s multiple comparisons test. CMG, cystometrogram; BP, bladder pressure.

We then evaluated potential treatment effects of direct intravesical infusion of imatinib in mice with acute CYP-induced cystitis. Mice were treated with imatinib via intrabladder infusion (50 μM, 0.5 mL, 30 min) *after* induction of acute (4 h) CYP cystitis (Figure 2A). Bladder function was recorded before and after imatinib infusion. Acute CYP treatment significantly (*p* ≤ 0.01) reduced IMI and IV in female mice (Figures 2B,D). Imatinib treatment significantly increased IMI and IV in female (1.7-fold) (*p* ≤ 0.0001; Figures 2B,D, 4A–D) and male (1.6-fold) (*p* ≤ 0.05; Figures 2C,E) mice with acute CYP-induced cystitis. Imatinib treatment significantly (*p* ≤ 0.05) reduced minimum bladder pressure in female mice with acute CYP-induced cystitis but did not affect bladder pressures in male mice (Table 2). Bladder function changes in mice with imatinib infusion were observed within 30–50 min. Most mice (n = 16/19, 84%) exhibited changes in bladder function following imatinib infusion that persisted until the end of the recording session (i.e., 30–60 min).

**TABLE 2:** Mean bladder pressures during conscious, open-outlet cystometry in control (no CYP), acute CYP (4 h or 24 h, 200 mg/kg, i.p.), or chronic CYP (75 mg/kg every 72 h for 8 days, i.p.) male and female mice treated with imatinib mesylate or vehicle (saline) via intravesical bladder infusion.

Group	Threshold pressure	Minimum pressure	Maximum pressure
*Female mice*			
Control (No CYP) Pre-Imatinib	26.3 ± 1.5	17.9 ± 1.9	33.8 ± 2.4
Post-Imatinib	26.5 ± 1.8	15.8 ± 1.4	32.8 ± 1.5
Acute CYP (4 h) Pre-Imatinib	28.9 ± 2.4	17.4 ± 1.7	39.4 ± 2.8
Post-Imatinib	27.0 ± 2.8	13.7 ± 0.8*	38.1 ± 2.8
Acute CYP (4 h) Pre-Imatinib Infusion #1	28.0 ± 3.1	18.1 ± 3.2	38.1 ± 2.8
Post-Imatinib Infusion #1	26.5 ± 3.4	16.5 ± 4.1	35.8 ± 3.4
24 h CYP Pre-Imatinib Infusion #2	29.1 ± 3.4	15.3 ± 2.2	29.1 ± 3.4
Post-Imatinib Infusion #2	29.3 ± 2.7	16.9 ± 2.1	29.3 ± 2.7
Chronic CYP (8 day) Pre-Imatinib	26.6 ± 2.6	16.0 ± 1.4	37.7 ± 2.3
Post-Imatinib	24.6 ± 2.2	15.6 ± 1.7	38.6 ± 2.4
*Male Mice*			
Control (No CYP) Pre-Imatinib	27.1 ± 1.4	18.7 ± 1.9	39.7 ± 2.4
Post-Imatinib	24.0 ± 1.6	16.4 ± 1.1	37.1 ± 1.7
Acute CYP (4 h) Pre-Imatinib	28.9 ± 4.2	24.2 ± 4.4	42.6 ± 4.3
Post-Imatinib	32.0 ± 6.6	25.6 ± 6.8	46.3 ± 6.8

n = 5–10. Values are means ± SEM by Student’s paired t-test (chronic CYP with imatinib infusion experiment) or repeated measures, two-way ANOVA with Bonferroni’s (4 h/24 h CYP with imatinib infusion experiment) or Šídák’s (female and male acute CYP with imatinib infusion experiment) multiple comparisons test. *p ≤ 0.05 when compared to 4 h CYP, pre-imatinib infusion.

### Imatinib Is Not Effective in Preventing or Treating Intermediate (48 h) or Chronic (8 Day) Cyclophosphamide-Induced Cystitis

**FIGURE 5:**
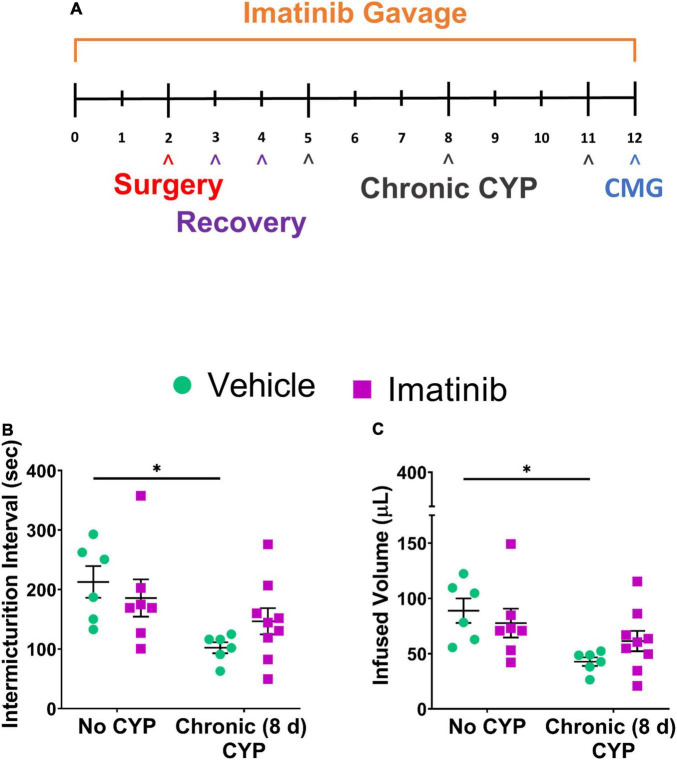
Imatinib mesylate pre-treatment via gavage does not significantly affect bladder function in mice with chronic (8 day) CYP-induced cystitis. **(A)** Chronic CYP-induced cystitis (75 mg/kg, every 72 h for 8 days) experimental schedule for imatinib mesylate pre-treatment via oral gavage (250 mg/kg, 1X/day). **(B,C)** Imatinib pre-treatment via gavage did not significantly affect IMI or IV in female mice with chronic CYP-induced cystitis. *n* = 7–9. Values are mean ± SEM by two-way ANOVA with Bonferroni’s multiple comparisons test. CMG, cystometrogram.

**FIGURE 6:**
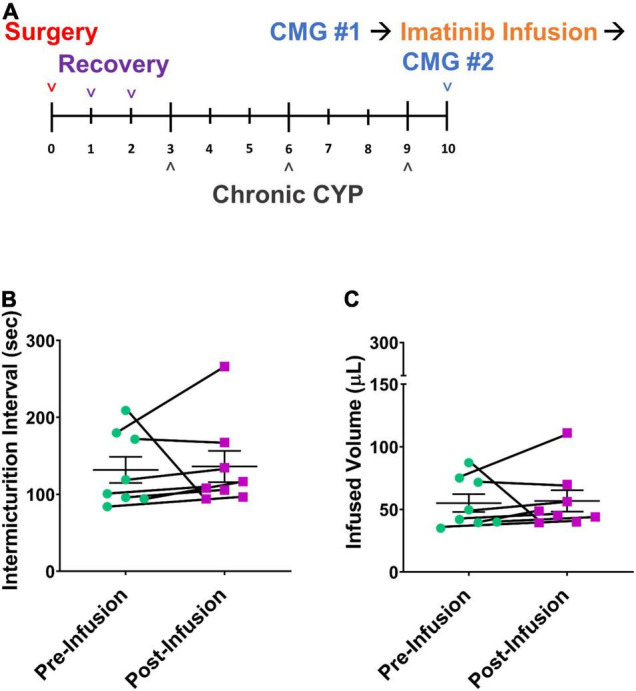
Imatinib mesylate treatment via intravesical infusion does not significantly affect bladder function in mice with chronic (8 day) CYP-induced cystitis. **(A)** Chronic CYP-induced cystitis (75 mg/kg, every 72 h for 8 days) experimental schedule for imatinib mesylate treatment via intravesical bladder infusion (50 μM, 30 min, 0.5 mL). **(B,C)** Imatinib treatment via intravesical bladder infusion did not significantly affect IMI or IV in chronic CYP-induced cystitis, female mice. *n* = 7–9. Values are mean ± SEM by Student’s paired *t*-test. CMG, cystometrogram.

We investigated the effects of PDGFRα blockade with imatinib in mice with intermediate (48 h) and chronic (8 day) CYP-induced cystitis using prevention (i.e., imatinib via oral gavage *before* CYP treatment) and treatment (i.e., CYP treatment *followed* by intrabladder infusion of imatinib) methods (Figures 5A, 6A and Supplementary Figure 3).

#### Prevention

Before CYP treatment, mice were pre-treated daily with imatinib for 5 days. CYP treatment began on the 5th day for intermediate and chronic schedules. Intermediate CYP treatment consisted of a single CYP injection (200 mg/kg, i.p.) followed by bladder function testing at 48 h (intermediate) after injection. For the chronic cystitis model, mice received 3 CYP injections every 72 h (75 mg/kg, i.p.) followed by bladder function testing on the last day. Daily imatinib administration continued throughout the CYP treatment period for a total of 7 or 12 total days for the intermediate or chronic CYP schedules, respectively. Mice with chronic (75 mg/kg, i.p. every 72 h, 8 days) CYP-induced cystitis exhibited significantly (*p* ≤ 0.05) decreased IMI and IV compared to controls (Figures 5B,C). Mice pre-treated with imatinib (250 mg/kg, 12 total days) by gavage with chronic CYP treatment, did not exhibit significant differences in IMI or IV compared to chronic CYP groups pre-treated with vehicle (Figures 5B,C). Imatinib pre-treatment significantly (*p* ≤ 0.01) increased minimum pressure in chronic CYP-induced cystitis female mice, compared to vehicle controls (Table 1). We additionally assessed the effectiveness of imatinib at an intermediate CYP-induced cystitis time point (200 mg/kg, i.p., 48 h) with imatinib pre-treatment (gavage, 250 mg/kg, 7 total days) and found no significant differences in IMI or IV compared to vehicle treated groups or controls (Supplementary Figure 3).

#### Treatment

Mice were treated by intravesical infusion with imatinib (50 μM, 0.5 mL, 30 min) on the last day of the chronic CYP protocol (Figure 6A; 75 mg/kg, i.p. every 72 h, 8 days). Bladder function was recorded before and after the imatinib intravesical infusion in the same mice. Mice with chronic CYP-induced cystitis treated with imatinib via intrabladder infusion did not exhibit significant differences in bladder function compared to pretreatment function (Figures 6B,C and Table 2).

Use of imatinib mesylate as a prevention or treatment for bladder dysfunction with CYP-induced cystitis was ineffective in intermediate (48 h) or chronic (8 day) CYP treatment protocols. Due to the absence of effect(s) in female mice, studies were not repeated with male cohorts.

### Oral Gavage of Imatinib Increases Voiding Frequency and Decreases Bladder Capacity in Control (No Cyclophosphamide) Mice

In prevention studies with imatinib pre-treatment, control (no CYP) groups were necessary and allowed us to assess the effect of PDGFRα inhibition on bladder function before CYP treatment. Control mice were treated with imatinib or vehicle throughout the experimental period (Figures 1A, 5A) and did not receive CYP injections. Female and male control (no CYP) mice treated with imatinib, via gavage, for the acute 5-day schedule, exhibited increased voiding frequency (decreased IMI and IV) (*p* ≤ 0.01), compared to vehicle treated control (no CYP) mice (Figures 1B–E). In female mice, decreases in IMI and IV were more dramatic in mice with acute CYP-induced cystitis treated with vehicle compared to the imatinib treated control (5 day) group (Figures 1B,D). However, in male mice, the imatinib treated control group (5 day) and 4 h CYP treatment with vehicle groups were not significantly different (Figures 1C,E). Imatinib significantly (*p* ≤ 0.01) increased bladder pressure for both female (threshold) and male (threshold and minimum) control mice (Table 1). Imatinib treatment in control mice (no CYP) did not significantly affect bladder function with the intermediate (8 day) (Supplementary Figure 3) or chronic (12 day) schedules (Figures 5B,C and Table 1).

### Intrabladder Infusion of Imatinib Decreases Voiding Frequency and Increases Bladder Capacity in Male and Female Control (No Cyclophosphamide) Mice

Imatinib treatment, via intrabladder infusion, significantly decreased voiding frequency (increased IMI and IV) in female (*p* ≤ 0.01) (1.5-fold) and male (*p* ≤ 0.05) (1.4-fold) control (no CYP) mice but did not affect bladder pressures in either sex (Figures 2B–E and Table 2).

### Multiple Treatments With Intrabladder Infusion of Imatinib May Further Improve Bladder Function With Acute (4 h) Cyclophosphamide-Induced Cystitis in Female Mice

**FIGURE 7:**
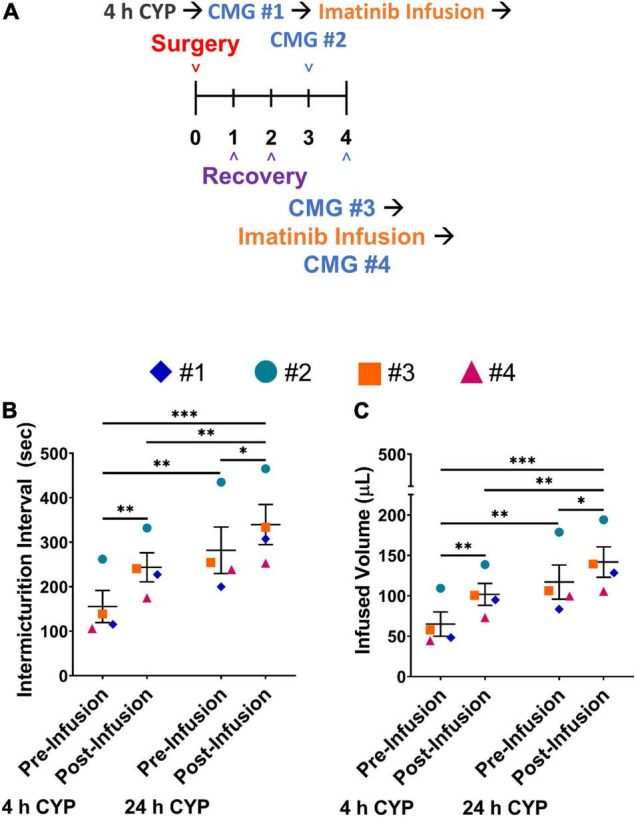
Two intravesical infusions with imatinib mesylate significantly increases IMI and IV in female mice with acute (4 h) CYP-induced cystitis. **(A)** Acute CYP-induced cystitis (200 mg/kg, 4 h, i.p.) experimental schedule for two-dose imatinib mesylate treatment via intravesical bladder infusion (50 μM, 30 min, 0.5 mL). Acute cystitis is induced on the third day. Bladder function with cystometrogram (CMG) is recorded 4 h after the CYP injection, before and after an intravesical bladder infusion of imatinib mesylate, and again, 24 h after the CYP injection on day 4, before and after a second imatinib mesylate intravesical bladder infusion. **(B,C)** Imatinib mesylate treatment significantly (*p* ≤ 0.01) increased the IMI and IV in female mice with acute CYP-induced cystitis at 4 and 24 h (*p* ≤ 0.001) following CYP. *n* = 4. Values are mean ± SEM. *p ≤ 0.05; ^**^p ≤ 0.01; ^***^p ≤ 0.001; by two-way repeated measures ANOVA with Bonferroni’s multiple comparisons test. CMG, cystometrogram.

We evaluated if an additional treatment with imatinib mesylate would result in a greater improvement in bladder function in the acute CYP-induced cystitis mouse model beyond what was observed with a single intrabladder infusion. In this experiment, mice received imatinib treatment (50 μM, 0.5 mL, 30 min) via intrabladder infusion 4 *and* 24 h after inducing acute (4 h) cystitis with CYP. Bladder function was recorded before and after imatinib treatment, both 4 and 24 h after CYP administration (Figure 7A). IMI and IV significantly increased (*p* ≤ 0.01) after the first dose of imatinib treatment with acute (4 h) CYP treatment (Figures 7B,C). Twenty-four hours later, before the second dose of imatinib, significant (*p* ≤ 0.01) changes in bladder function (e.g., increased IMI and IV) persisted. The second dose of imatinib, 24 h after CYP treatment significantly (*p* ≤ 0.05) increased IMI and IV compared to acute (4 h) CYP-induced cystitis before (*p* ≤ 0.001) and after the effects of a single dose of imatinib (*p* ≤ 0.01; Figures 7B,C). Imatinib treatment did not affect bladder pressures at 4 or 24 h (Table 2). These results suggest that multiple doses of imatinib may improve bladder function outcomes with acute (4 h) CYP-induced cystitis.

### Oral Gavage or Transurethral Infusion of Imatinib Does Not Affect Pelvic Somatic Sensitivity in Female Mice With Acute (4 h) Cyclophosphamide-Induced Cystitis

Acute CYP administration significantly (*p* ≤ 0.05) increased pelvic somatic sensitivity compared to control (no CYP) (Supplementary Figures 1, 2). In controls (no CYP), imatinib delivery via gavage (prevention protocol) increased pelvic sensitivity (*p* ≤ 0.05) but no effect was observed with transurethral infusion of imatinib (treatment protocol) (Supplementary Figures 1, 2). In mice with acute CYP-induced cystitis, imatinib treatment (transurethral intrabladder infusion, 50 μM, 0.5 mL, 30 min) or prevention (oral gavage, 250 mg/kg, 5 days) did not affect pelvic somatic sensitivity (Supplementary Figures 1, 2). We additionally scored behaviors in female mice with acute CYP-induced cystitis following transurethral infusion (50 μM, 0.5 mL, 30 min) of imatinib or vehicle. Total licking behaviors were significantly (p ≤ 0.05) increased in mice with 4 h CYP-induced cystitis. However, imatinib treatment did not significantly affect either total time moving or total licking behaviors in control mice or in mice with 4 h CYP-induced cystitis (Supplementary Figure 2).

## Discussion

Our studies demonstrate different effects of imatinib treatment depending on its use in control (no CYP), CYP-treated or mice with varying durations of CYP-treatment. Our studies expand upon previous reports investigating the effects of imatinib in the micturition system in a variety of bladder dysfunction models, including: CYP-induced cystitis (Sancho et al., 2017; Liu et al., 2018), radiation-induced cystitis (Giglio et al., 2018); spinal cord injury (SCI)-induced overactive bladder (Abrams et al., 2012; Deng et al., 2013; Kjell et al., 2015), and bladder outlet obstruction (Kim et al., 2011; Preis et al., 2015). Although various *in vivo* and *ex vivo* approaches have been used to determine effects of imatinib on rat and guinea pig bladder function (Kubota et al., 2004, 2006; Biers et al., 2006; Kim et al., 2011; Abrams et al., 2012; Deng et al., 2013; Gevaert et al., 2014a; Kjell et al., 2015; Sancho et al., 2017; Giglio et al., 2018; Liu et al., 2018), we believe these studies are the first to use conscious, unrestrained cystometry to assess the effects of imatinib mesylate on urinary bladder function in mice with CYP-induced cystitis. Our results suggest imatinib prevention and treatment improve LUT storage outcomes in female mice with acute (4 h) CYP-induced cystitis but not chronic CYP-induced cystitis. Interestingly, our prevention (gavage) studies in control mice (no CYP) also demonstrate that imatinib treatment can increase voiding frequency suggesting that imatinib and PDGFRα+ interstitial cells may contribute to the development and maintenance of bladder dysfunction (e.g., increased voiding frequency). Although cystometry results demonstrate that intravesical or oral imatinib can improve urinary bladder function in acute (4 h) CYP cystitis, imatinib does not affect mechanical sensitivity of the pelvic region in these mice. The absence of effect of imatinib on mechanical sensitivity may suggest that imatinib will not effectively treat pain symptoms in IC/BPS patients but could be useful for OAB patients. Ultimately, our studies suggest imatinib mesylate given systemically (gavage) or intravesically as a potential therapeutic for LUT storage symptoms in IC/BPS and/or OAB. Imatinib prevention (i.e., gavage, 250 mg/kg, 1X/day, 5 days) increased IV and IMI in female, but not male, mice with acute CYP-induced cystitis. Although imatinib does inhibit the tyrosine kinase receptor PDGFRα, like the majority of other PDGFRα inhibitors, it is not specific (Roskoski, 2018). Imatinib additionally inhibits PDGFRβ, c-KIT and BCR-ABL (Buchdunger et al., 2000; Capdeville et al., 2002). With systemic administration of imatinib, we cannot exclude the possibility that improvements in bladder outcomes were due to blockade of PDGFRα and other receptors throughout the body. Although we are unable to achieve specific PDGFRα inhibition with imatinib given systemically or intravesically, we used intravesical delivery to target the site of action of imatinib to the urinary bladder.

For the intravesical imatinib administration experiments, we initially conducted a preventative design with daily imatinib bladder infusions *prior* to induction of acute (4 h) CYP-induced cystitis on the last experimental day. However, imatinib prevention via intravesical infusion did not affect LUT outcomes in acute CYP-induced cystitis mice. We next evaluated intravesical treatment studies to examine the effect of imatinib on bladder function *after* inducing acute CYP cystitis. Intravesical imatinib treatment significantly improved LUT function in female and male mice with acute CYP-induced cystitis. Imatinib prevention and treatment had similar magnitude of effect in increasing IMI and IV in CYP-treated mice. Despite improvements in LUT outcomes, imatinib prevention was associated with increases in bladder pressures, while intravesical infusion of imatinib reduced or had no effect on bladder pressures. If these data can eventually be translated to humans with IC/BPS, the magnitude of effect with route of administration, the ease and convenience of administration, as well as off-target effects will need to be addressed.

Surprisingly, imatinib prevention in control (no CYP) mice increased voiding frequency compared to vehicle treated controls. However, the increased voiding frequency in female controls was less than that observed in female mice with acute CYP-induced cystitis. Increased voiding frequency in control mice treated with systemic (gavage) imatinib treatment may be, in part, due to PDGFRα inhibition of detrusor interstitial cells. Our studies did not directly assess the contribution of detrusor interstitial cells to the increased voiding frequency observed in control mice pre-treated with imatinib. However, studies show that PDGFRα+ detrusor interstitial cells exhibit inhibitory influence over smooth muscle cells (SMCs) to regulate contractions during bladder filling, likely via SK3 channels (Parajuli et al., 2012; Anderson et al., 2013; Lee et al., 2013, 2017; Koh et al., 2018). SK3 agonists enhance outward currents in PDGFRα+ detrusor interstitial cells and reduce detrusor contractions, whereas antagonists block outward currents and increase contractions (Lee et al., 2013, 2014, 2017). Thus, the increased voiding frequency in control mice pre-treated with imatinib may be due imatinib inhibition of PDGFRα+ detrusor interstitial cells, resulting in the release of interstitial cell inhibitory control over detrusor SMCs to produce an increase in voiding frequency. Interestingly, similar results were not observed when control mice were treated with intravesical imatinib administration. Imatinib treatment (50 μM, 0.5 mL, 30 min) increased bladder capacity and reduced voiding frequency in control (non-CYP) female and male mice. These results may, in part, be due to imatinib blockade of PDGFRα expressed by LP interstitial cells. Although we cannot discount an effect of imatinib on detrusor interstitial cells with the treatment design, intravesical infusion of imatinib would likely first act on LP interstitial cells based upon their proximity to the bladder lumen infusion site. As suggested, LP interstitial cells may act as sensory intermediaries to the detrusor and/or afferent nerves (Wiseman et al., 2003; Gray et al., 2013; Heppner et al., 2017; Sancho et al., 2017; Koh et al., 2018). It may be possible that blockade of LP interstitial cells disrupts communication with bladder afferent nerves, resulting in dysregulated sensory signaling, and in turn, increased IMI and IV. However, these ideas should be further researched as our studies do not directly assess the contribution of specific interstitial cells to LUT function in the control or inflamed bladder.

Imatinib was effective at improving bladder function in mice with acute (4 h) CYP-induced cystitis for both the prevention (systemic gavage) and treatment (intravesical) designs, yet imatinib did not affect bladder function in mice with intermediate (48 h; gavage) or chronic (8 day; gavage and intravesical) CYP-induced cystitis. Many significant changes occur throughout the micturition system (e.g., inflammatory mediator expression, peripheral and central sensitization) which contribute to symptom progression and exacerbation with acute, intermediate, and chronic CYP-induced cystitis (Malley and Vizzard, 2002; Arms et al., 2013; Gonzalez et al., 2014; Guo et al., 2018). These nuances increase the complexity of treating urinary bladder dysfunction and may suggest that some treatments may only be effective at certain timepoints. In addition, a combination of therapeutics and treatments may be beneficial when treating the progression of acute to chronic urinary bladder inflammation. Future studies in mice with acute, intermediate, and chronic CYP-induced cystitis that evaluate bladder function effects when imatinib is combined with other treatments (e.g., neuropeptide, cytokine, and chemokine receptor antagonists) should be considered (Arms et al., 2010, 2013; Gonzalez et al., 2013, 2014, 2016; Merrill and Vizzard, 2014; Girard et al., 2016, 2019, 2021; Guo et al., 2018; Tooke et al., 2019). In addition, our results assessing imatinib treatment at 4 and 24 h after CYP treatment, suggest repeated use of imatinib may offer additional improvements in bladder function in acute CYP-induced cystitis and extend symptom relief. Future studies should determine the duration of symptom relief period and if bladder dysfunction can be fully reversed with multiple imatinib treatments.

In addition to LUT symptoms, IC/BPS patients report unpleasant pelvic sensations (e.g., pain, pressure, or discomfort) (Hanno et al., 2015). Thus, we also examined mechanical sensitivity of the abdominal/pelvic region in cystitis with imatinib administration. Our results suggest that imatinib prevention and treatment improves functional bladder outcomes (i.e., IV, IMI), but may not affect pelvic sensation. If clinically translatable to human patients, these results may suggest that imatinib will not effectively treat the pelvic pain component of IC/BPS and that imatinib may be a more suitable treatment for OAB. The therapeutic potential of imatinib for OAB warrants further research as our studies did not assess the effect of imatinib on LUT function in an OAB model. The differential effects on bladder function and pelvic sensitivity were surprising to us because we have evaluated multiple pharmacological agents (Arms et al., 2013; Girard et al., 2016, 2019, 2021; Guo et al., 2018) in rodents with CYP-induced cystitis and have never demonstrated improvements in bladder function *without* reductions in mechanical sensitivity. However, these results may provide information about the role of LP interstitial cells in the urinary bladder and their potential communication with bladder nerve fibers. Additional studies are planned to evaluate the effect of imatinib on pelvic sensitivity in CYP-induced cystitis using visceromotor responses.

Overexpression and enhanced activity of PDGFs/Rs may indicate pathological processes (e.g., cancerous, fibrotic, inflammatory, and vascular conditions) (Heldin and Westermark, 1999; Andrae et al., 2008). In urinary bladder dysfunction, PDGFs/Rs may contribute to underlying mechanisms that induce or sustain inflammation. Although the PDGFRα LP interstitial cells likely serve important roles in the normal functioning bladder (e.g., communicating bladder fullness to afferent nerves and regulating detrusor muscle contractions) (Heppner et al., 2017), they may also contribute to pathological states by altering the inflammatory milieu of the urinary bladder. In mice with incomplete SCI treated with imatinib mesylate, serum levels of monocyte chemoattractant protein-1, macrophage inflammatory protein (MIP)-3α, and keratinocyte chemoattractant/growth-regulated oncogene (interleukin 8) were significantly increased (Kjell et al., 2015) and yet mice exhibited improvements in bladder function and hindlimb locomotion. Future studies that address mechanisms underlying bladder functional recovery in the presence of, or possibly supported by, an inflammatory activation by imatinib are needed. We are currently examining inflammatory activation in the LUT by imatinib in mice with and without CYP-induced cystitis.

In conclusion, our results suggest imatinib administration improves bladder function (e.g., IMI and IV) in mice with acute CYP-induced cystitis by a prevention (gavage; females) and treatment (intravesical infusion; females and males) experimental design. Bladder function improvements in the acute CYP-induced cystitis model with imatinib administration may be due, at least in part, to inhibition of the PDGFRα+ LP interstitial cells. Furthermore, these studies demonstrated changes in voiding frequency with imatinib administration supporting the suggestion that these cells function as a sensory intermediary role in control as well as the inflamed urinary bladder. The absence of effect of imatinib administration by the prevention (gavage) and treatment (intravesical infusion) on mechanical sensitivity in mice with acute CYP-induced cystitis may suggest that imatinib mesylate may be more appropriate as a treatment for OAB where patients present with urinary storage symptoms (e.g., increased urgency, frequency, nocturia) without pelvic pain, pressure or discomfort reported by IC/BPS patients (Macdiarmid and Sand, 2007). These studies provide some insight into a novel cell population in the urinary bladder that may be an effective target in managing cystitis-induced bladder dysfunction.

## Data Availability Statement

The original contributions presented in the study are included in the article/Supplementary Material, further inquiries can be directed to the corresponding author.

## Ethics Statement

The animal study was reviewed and approved by the University of Vermont Institutional Animal Care and Use Committee.

## Author Contributions

MP, BG, and MV conceived and designed the research, interpreted the results of experiments, and edited and revised the manuscript. MP, SC, and BG performed the experiments. MP analyzed the data, prepared the figures, and drafted the manuscript. MP, BG, SC, and MV approved the final version of manuscript. All authors contributed to the article and approved the submitted version.

## Author Disclaimer

The National Institutes of Health had no role in the experiments described, including the design, data collection, and analysis of studies performed in the Vizzard laboratory, decision to publish, or preparation of the manuscript. The contents are solely the responsibility of the authors and do not necessarily represent the official views of the National Institutes of Health.

## Conflict of Interest

The authors declare that the research was conducted in the absence of any commercial or financial relationships that could be construed as a potential conflict of interest.

## Publisher’s Note

All claims expressed in this article are solely those of the authors and do not necessarily represent those of their affiliated organizations, or those of the publisher, the editors and the reviewers. Any product that may be evaluated in this article, or claim that may be made by its manufacturer, is not guaranteed or endorsed by the publisher.

## Footnotes

1
www.graphpad.com

## Abbreviations

IC/BPS: interstitial cystitis/bladder pain syndrome
OAB: overactive bladder
SCI: spinal cord injury
LUT: lower urinary tract
WT: wild type
PDGFRα: platelet-derived growth factor receptor alpha
PDGF: platelet-derived growth factors
c-KIT: tyrosine-protein kinase KIT
CML: chronic myeloid leukemia
CYP: cyclophosphamide
PS: protamine sulfate
i.p.: intraperitoneal
s.c.: subcutaneous
LP: lamina propria
DRG: dorsal root ganglia
SMC: smooth muscle cell
ATP: adenosine triphosphate
ACh: acetylcholine
CCL2: C-C motif chemokine ligand 2
MAPK/ERK: mitogen-activated protein kinase/extracellular signal-related kinase
PI3K/AKT: phosphatidylinositol 3-kinase/protein kinase B
PLC-γ: phospholipase C-GAMMA
IMI: intermicturition interval
IV: infused volume
BP: bladder pressure
NVC: non-voiding contraction
h: hour(s)
min: minutes
s or sec: seconds
ANOVA: analysis of variance
CMG: cystometrogram.

## References

[B1] Abrams M. B. Nilsson I. Lewandowski S. A. Kjell J. Codeluppi S. Olson L. (2012). Imatinib enhances functional outcome after spinal cord injury. *PLoS One* 7:e38760. 10.1371/journal.pone.0038760 22723886 PMC3378614

[B2] Anderson U. A. Carson C. Johnston L. Joshi S. Gurney A. M. McCloskey K. D. (2013). Functional expression of KCNQ (Kv7) channels in guinea pig bladder smooth muscle and their contribution to spontaneous activity. *Br. J. Pharmacol.* 169 1290–1304. 10.1111/bph.12210 23586426 PMC3746117

[B3] Andersson K. E. (2002). Bladder activation: afferent mechanisms. *Urology* 59(5 Suppl. 1), 43–50. 10.1016/s0090-4295(01)01637-5 12007522

[B4] Andersson K. E. (2004). Mechanisms of Disease: central nervous system involvement in overactive bladder syndrome. *Nat. Clin. Pract. Urol.* 1 103–108. 10.1038/ncpuro0021 16474523

[B5] Andrae J. Gallini R. Betsholtz C. (2008). Role of platelet-derived growth factors in physiology and medicine. *Genes Dev.* 22 1276–1312. 10.1101/gad.1653708 18483217 PMC2732412

[B6] Arms L. Girard B. M. Malley S. E. Vizzard M. A. (2013). Expression and function of CCL2/CCR2 in rat micturition reflexes and somatic sensitivity with urinary bladder inflammation. *Am. J. Physiol. Renal Physiol.* 305 F111–F122. 10.1152/ajprenal.00139.2013 23594826 PMC3725675

[B7] Arms L. Girard B. M. Vizzard M. A. (2010). Expression and function of CXCL12/CXCR4 in rat urinary bladder with cyclophosphamide-induced cystitis. *Am. J. Physiol. Renal Physiol.* 298 F589–F600. 10.1152/ajprenal.00628.2009 20032115 PMC2838600

[B8] Arras M. Rettich A. Cinelli P. Kasermann H. P. Burki K. (2007). Assessment of post-laparotomy pain in laboratory mice by telemetric recording of heart rate and heart rate variability. *BMC Vet. Res.* 3:16. 10.1186/1746-6148-3-16 17683523 PMC1965463

[B9] Biers S. M. Reynard J. M. Doore T. Brading A. F. (2006). The functional effects of a c-kit tyrosine inhibitor on guinea-pig and human detrusor. *BJU Int.* 97 612–616. 10.1111/j.1464-410X.2005.05988.x 16469036

[B10] Birder L. Andersson K. E. (2013). Urothelial signaling. *Physiol. Rev.* 93 653–680. 10.1152/physrev.00030.2012 23589830 PMC3768101

[B11] Birder L. A. (2005). More than just a barrier: urothelium as a drug target for urinary bladder pain. *Am. J. Physiol. Renal Physiol.* 289 F489–F495. 10.1152/ajprenal.00467.2004 16093424

[B12] Bjorling D. E. Wang Z. Y. Bushman W. (2011). Models of inflammation of the lower urinary tract. *Neurourol. Urodyn.* 30 673–682. 10.1002/nau.21078 21661012 PMC3113627

[B13] Boucher M. Meen M. Codron J. P. Coudore F. Kemeny J. L. Eschalier A. (2000). Cyclophosphamide-induced cystitis in freely-moving conscious rats: behavioral approach to a new model of visceral pain. *J. Urol.* 164 203–208. 10840460

[B14] Buchdunger E. Cioffi C. L. Law N. Stover D. Ohno-Jones S. Druker B. J. (2000). Abl protein-tyrosine kinase inhibitor STI571 inhibits *in vitro* signal transduction mediated by c-kit and platelet-derived growth factor receptors. *J. Pharmacol. Exp. Ther.* 295 139–145. 10991971

[B15] Buchdunger E. Zimmermann J. Mett H. Meyer T. Müller M. Druker B. J. (1996). Inhibition of the Abl protein-tyrosine kinase *in vitro* and *in vivo* by a 2-phenylaminopyrimidine derivative. *Cancer Res.* 56 100–104. 8548747

[B16] Capdeville R. Buchdunger E. Zimmermann J. Matter A. (2002). Glivec (STI571, imatinib), a rationally developed, targeted anticancer drug. *Nat. Rev. Drug Discov.* 1 493–502. 10.1038/nrd839 12120256

[B17] Chuang Y. C. Chancellor M. B. Seki S. Yoshimura N. Tyagi P. Huang L. (2003). Intravesical protamine sulfate and potassium chloride as a model for bladder hyperactivity. *Urology* 61 664–670. 10.1016/s0090-4295(02)02280-x 12639680

[B18] Dalghi M. G. Clayton D. R. Ruiz W. G. Al-Bataineh M. M. Satlin L. M. Kleyman T. R. (2019). Expression and distribution of PIEZO1 in the mouse urinary tract. *Am. J. Physiol. Renal Physiol.* 317 F303–F321. 10.1152/ajprenal.00214.2019 31166705 PMC6732449

[B19] de Groat W. C. Griffiths D. Yoshimura N. (2015). Neural control of the lower urinary tract. *Compr. Physiol.* 5 327–396. 10.1002/cphy.c130056 25589273 PMC4480926

[B20] de Jongh R. van Koeveringe G. A. van Kerrebroeck P. E. Markerink-van Ittersum M. de Vente J. Gillespie J. I. (2007). Alterations to network of NO/cGMP-responsive interstitial cells induced by outlet obstruction in guinea-pig bladder. *Cell Tissue Res.* 330 147–160. 10.1007/s00441-007-0454-y 17710439

[B21] Deng J. Zhang Y. Wang L. Zhao J. Song B. Li L. (2013). The effects of Glivec on the urinary bladder excitation of rats with suprasacral or sacral spinal cord transection. *J. Surg. Res.* 183 598–605. 10.1016/j.jss.2013.02.030 23608618

[B22] Deng T. Zhang Q. Wang Q. Zhong X. Li L. (2015). Changes in hyperpolarization-activated cyclic nucleotide-gated channel expression and activity in bladder interstitial cells of Cajal from rats with detrusor overactivity. *Int. Urogynecol. J.* 26 1139–1145. 10.1007/s00192-015-2632-x 25677606

[B23] Deuis J. R. Dvorakova L. S. Vetter I. (2017). Methods used to evaluate pain behaviors in rodents. *Front. Mol. Neurosci.* 10:284. 10.3389/fnmol.2017.00284 28932184 PMC5592204

[B24] Fraser M. O. Chuang Y. C. Tyagi P. Yokoyama T. Yoshimura N. Huang L. (2003). Intravesical liposome administration–a novel treatment for hyperactive bladder in the rat. *Urology* 61 656–663. 10.1016/s0090-4295(02)02281-1 12639679

[B25] Fry C. H. Young J. S. Jabr R. I. McCarthy C. Ikeda Y. Kanai A. J. (2012). Modulation of spontaneous activity in the overactive bladder: the role of P2Y agonists. *Am. J. Physiol. Renal Physiol.* 302 F1447–F1454. 10.1152/ajprenal.00436.2011 22357922 PMC3378171

[B26] Gabella G. (2019). Afferent nerve fibres in the wall of the rat urinary bladder. *Cell Tissue Res.* 376 25–35. 10.1007/s00441-018-2965-0 30523406

[B27] Gevaert T. De Vos R. Everaerts W. Libbrecht L. Van Der Aa F. van den Oord J. (2011). Characterization of upper lamina propria interstitial cells in bladders from patients with neurogenic detrusor overactivity and bladder pain syndrome. *J. Cell. Mol. Med.* 15 2586–2593. 10.1111/j.1582-4934.2011.01262.x 21251216 PMC4373427

[B28] Gevaert T. Hutchings G. Everaerts W. Prenen H. Roskams T. Nilius B. (2014a). Administration of imatinib mesylate in rats impairs the neonatal development of intramuscular interstitial cells in bladder and results in altered contractile properties. *Neurourol. Urodyn.* 33 461–468. 10.1002/nau.22415 23616342

[B29] Gevaert T. Vanstreels E. Daelemans D. Franken J. Van Der Aa F. Roskams T. (2014b). Identification of different phenotypes of interstitial cells in the upper and deep lamina propria of the human bladder dome. *J. Urol.* 192 1555–1563. 10.1016/j.juro.2014.05.096 24893312

[B30] Gevaert T. Moles Lopez X. Sagaert X. Libbrecht L. Roskams T. Rorive S. (2015). Morphometric and quantitative immunohistochemical analysis of disease-related changes in the upper (suburothelial) lamina propria of the human bladder dome. *PLoS One* 10:e0127020. 10.1371/journal.pone.0127020 25973881 PMC4431865

[B31] Gevaert T. Ridder D. Vanstreels E. Daelemans D. Everaerts W. Aa F. V. (2017). The stem cell growth factor receptor KIT is not expressed on interstitial cells in bladder. *J. Cell. Mol. Med.* 21 1206–1216. 10.1111/jcmm.13054 27997763 PMC5431123

[B32] Giglio D. Podmolíková L. Tobin G. (2018). Changes in the neuronal control of the urinary bladder in a model of radiation cystitis. *J. Pharmacol. Exp. Ther.* 365 327–335. 10.1124/jpet.117.246371 29530925

[B33] Gillespie J. I. Markerink-van Ittersum M. De Vente J. (2006). Endogenous nitric oxide/cGMP signalling in the guinea pig bladder: evidence for distinct populations of sub-urothelial interstitial cells. *Cell Tissue Res.* 325 325–332. 10.1007/s00441-005-0146-4 16598501

[B34] Girard B. M. Campbell S. E. Beca K. I. Perkins M. Hsiang H. May V. (2021). Intrabladder PAC1 Receptor Antagonist, PACAP(6-38), reduces urinary bladder frequency and pelvic sensitivity in mice exposed to repeated Variate Stress (RVS). *J. Mol. Neurosci.* 71 1575–1588. 10.1007/s12031-020-01649-x 32613552 PMC7775277

[B35] Girard B. M. Campbell S. E. Perkins M. Hsiang H. Tooke K. Drescher C. (2019). TRPV4 blockade reduces voiding frequency, ATP release, and pelvic sensitivity in mice with chronic urothelial overexpression of NGF. *Am. J. Physiol. Renal Physiol.* 317 F1695–F1706. 10.1152/ajprenal.00147.2019 31630542 PMC6962511

[B36] Girard B. M. Malley S. E. Mathews M. M. May V. Vizzard M. A. (2016). Intravesical PAC1 receptor antagonist, PACAP(6-38), reduces urinary bladder frequency and pelvic sensitivity in NGF-OE Mice. *J. Mol. Neurosci.* 59 290–299. 10.1007/s12031-016-0764-1 27146136 PMC5252828

[B37] Gonzalez E. J. Arms L. Vizzard M. A. (2014). The role(s) of cytokines/chemokines in urinary bladder inflammation and dysfunction. *Biomed Res. Int.* 2014:120525. 10.1155/2014/120525 24738044 PMC3971501

[B38] Gonzalez E. J. Girard B. M. Vizzard M. A. (2013). Expression and function of transforming growth factor-β isoforms and cognate receptors in the rat urinary bladder following cyclophosphamide-induced cystitis. *Am. J. Physiol. Renal Physiol.* 305 F1265–F1276. 10.1152/ajprenal.00042.2013 23926183 PMC3840223

[B39] Gonzalez E. J. Heppner T. J. Nelson M. T. Vizzard M. A. (2016). Purinergic signalling underlies transforming growth factor-β-mediated bladder afferent nerve hyperexcitability. *J. Physiol.* 594 3575–3588. 10.1113/JP272148 27006168 PMC4929319

[B40] Gray S. M. McGeown J. G. McMurray G. McCloskey K. D. (2013). Functional innervation of Guinea-pig bladder interstitial cells of cajal subtypes: neurogenic stimulation evokes in situ calcium transients. *PLoS One* 8:e53423. 10.1371/journal.pone.0053423 23326426 PMC3541194

[B41] Grol S. Essers P. B. van Koeveringe G. A. Martinez-Martinez P. de Vente J. Gillespie J. I. M. (2009). (3) muscarinic receptor expression on suburothelial interstitial cells. *BJU Int.* 104 398–405. 10.1111/j.1464-410X.2009.08423.x 19338557

[B42] Guo M. Chang P. Hauke E. Girard B. M. Tooke K. Ojala J. (2018). Expression and Function of Chemokines CXCL9-11 in micturition pathways in cyclophosphamide (CYP)-induced cystitis and somatic sensitivity in mice. *Front. Syst. Neurosci.* 12:9. 10.3389/fnsys.2018.00009 29681802 PMC5897511

[B43] Hanno P. M. Erickson D. Moldwin R. Faraday M. M. (2015). American Urological Association. Diagnosis and treatment of interstitial cystitis/bladder pain syndrome: AUA guideline amendment. *J. Urol.* 193 1545–1553. 10.1016/j.juro.2015.01.086 25623737

[B44] Heldin C. H. Westermark B. (1999). Mechanism of action and in vivo role of platelet-derived growth factor. *Physiol. Rev.* 79 1283–1316. 10.1152/physrev.1999.79.4.1283 10508235

[B45] Heppner T. J. Hennig G. W. Nelson M. T. Vizzard M. A. (2017). Rhythmic calcium events in the lamina propria network of the urinary bladder of rat pups. *Front. Syst. Neurosci.* 11:87. 10.3389/fnsys.2017.00087 29321730 PMC5732214

[B46] Ikeda Y. Fry C. Hayashi F. Stolz D. Griffiths D. Kanai A. (2007). Role of gap junctions in spontaneous activity of the rat bladder. *Am. J. Physiol. Renal Physiol.* 293 F1018–F1025. 10.1152/ajprenal.00183.2007 17581924 PMC3037091

[B47] Isogai A. Lee K. Mitsui R. Hashitani H. (2016). Functional coupling of TRPV4 channels and BK channels in regulating spontaneous contractions of the guinea pig urinary bladder. *Pflugers Arch.* 468 1573–1585. 10.1007/s00424-016-1863-0 27497848

[B48] Johnston L. Cunningham R. M. Young J. S. Fry C. H. McMurray G. Eccles R. (2012). Altered distribution of interstitial cells and innervation in the rat urinary bladder following spinal cord injury. *J. Cell. Mol. Med.* 16 1533–1543. 10.1111/j.1582-4934.2011.01410.x 21883887 PMC3823221

[B49] Kanai A. Roppolo J. Ikeda Y. Zabbarova I. Tai C. Birder L. (2007). Origin of spontaneous activity in neonatal and adult rat bladders and its enhancement by stretch and muscarinic agonists. *Am. J. Physiol. Renal Physiol.* 292 F1065–F1072. 10.1152/ajprenal.00229.2006 17107944 PMC3033037

[B50] Kilic T. Alberta J. A. Zdunek P. R. Acar M. Iannarelli P. O’Reilly T. (2000). Intracranial inhibition of platelet-derived growth factor-mediated glioblastoma cell growth by an orally active kinase inhibitor of the 2-phenylaminopyrimidine class. *Cancer Res.* 60 5143–5150. 11016641

[B51] Kim S. O. Oh B. S. Chang I. Y. Song S. H. Ahn K. Hwang E. C. (2011). Distribution of interstitial cells of Cajal and expression of nitric oxide synthase after experimental bladder outlet obstruction in a rat model of bladder overactivity. *Neurourol. Urodyn.* 30 1639–1645. 10.1002/nau.21144 21780165

[B52] Kjell J. Finn A. Hao J. Wellfelt K. Josephson A. Svensson C. I. (2015). Delayed imatinib treatment for acute spinal cord injury: functional recovery and serum biomarkers. *J. Neurotrauma* 32 1645–1657. 10.1089/neu.2014.3863 25914996 PMC4752188

[B53] Klinger M. B. Vizzard M. A. (2008). Role of p75NTR in female rat urinary bladder with cyclophosphamide-induced cystitis. *Am. J. Physiol. Renal Physiol.* 295 F1778–F1789. 10.1152/ajprenal.90501.2008 18842820 PMC2604835

[B54] Koh B. H. Roy R. Hollywood M. A. Thornbury K. D. McHale N. G. Sergeant G. P. (2012). Platelet-derived growth factor receptor-α cells in mouse urinary bladder: a new class of interstitial cells. *J. Cell Mol. Med.* 16 691–700. 10.1111/j.1582-4934.2011.01506.x 22151424 PMC3822840

[B55] Koh S. D. Lee H. Ward S. M. Sanders K. M. (2018). The mystery of the interstitial cells in the Urinary Bladder. *Annu. Rev. Pharmacol. Toxicol.* 58 603–623. 10.1146/annurev-pharmtox-010617-052615 28992432

[B56] Kubota Y. Biers S. M. Kohri K. Brading A. F. (2006). Effects of imatinib mesylate (Glivec) as a c-kit tyrosine kinase inhibitor in the guinea-pig urinary bladder. *Neurourol. Urodyn.* 25 205–210. 10.1002/nau.20085 16425211

[B57] Kubota Y. Hashitani H. Shirasawa N. Kojima Y. Sasaki S. Mabuchi Y. (2008). Altered distribution of interstitial cells in the guinea pig bladder following bladder outlet obstruction. *Neurourol. Urodyn.* 27 330–340. 10.1002/nau.20502 17724735

[B58] Kubota Y. Kajioka S. Biers S. M. Yokota E. Kohri K. Brading A. F. (2004). Investigation of the effect of the c-kit inhibitor Glivec on isolated guinea-pig detrusor preparations. *Auton. Neurosci.* 115 64–73. 10.1016/j.autneu.2004.08.004 15507407

[B59] Lee H. Koh B. H. Peri L. E. Corrigan R. D. Lee H. T. George N. E. (2017). Premature contractions of the bladder are suppressed by interactions between TRPV4 and SK3 channels in murine detrusor PDGFRα^+^ cells. *Sci. Rep.* 7:12245. 10.1038/s41598-017-12561-7 28947806 PMC5613012

[B60] Lee H. Koh B. H. Peri L. E. Sanders K. M. Koh S. D. (2013). Functional expression of SK channels in murine detrusor PDGFR+ cells. *J. Physiol.* 591 503–513. 10.1113/jphysiol.2012.241505 23148317 PMC3577524

[B61] Lee H. Koh B. H. Peri L. E. Sanders K. M. Koh S. D. (2014). Purinergic inhibitory regulation of murine detrusor muscles mediated by PDGFRα+ interstitial cells. *J. Physiol.* 592 1283–1293. 10.1113/jphysiol.2013.267989 24396055 PMC3961087

[B62] Liu Q. Long Z. Dong X. Zhang T. Zhao J. Sun B. (2017). Cyclophosphamide-induced HCN1 channel upregulation in interstitial Cajal-like cells leads to bladder hyperactivity in mice. *Exp. Mol. Med.* 49:e319. 10.1038/emm.2017.31 PMC6130216 28428632

[B63] Liu Q. Sun B. Zhao J. Wang Q. An F. Hu X. (2018). Increased Piezo1 channel activity in interstitial Cajal-like cells induces bladder hyperactivity by functionally interacting with NCX1 in rats with cyclophosphamide-induced cystitis. *Exp. Mol. Med.* 50 1–16. 10.1038/s12276-018-0088-z PMC5938236 29735991

[B64] Macdiarmid S. A. Sand P. K. (2007). Diagnosis of interstitial cystitis/painful bladder syndrome in patients with overactive bladder symptoms. *Rev. Urol.* 9 9–16. 17396167 PMC1832106

[B65] Malley S. E. Vizzard M. A. (2002). Changes in urinary bladder cytokine mRNA and protein after cyclophosphamide-induced cystitis. *Physiol. Genomics* 9 5–13. 10.1152/physiolgenomics.00117.2001 11948286

[B66] May V. Vizzard M. A. (2010). Bladder dysfunction and altered somatic sensitivity in PACAP-/- mice. *J. Urol.* 183 772–779. 10.1016/j.juro.2009.09.077 20022034 PMC2917789

[B67] Meng M. Zheng J. Yan J. Li Q. Fang Q. Li W. (2015). P2X2 and P2X5 receptors mediate bladder hyperesthesia in ICC in female overactive bladder. *Cell Biochem. Biophys.* 72 375–383. 10.1007/s12013-014-0471-x 25561285

[B68] Merrill L. Gonzalez E. J. Girard B. M. Vizzard M. A. (2016). Receptors, channels, and signalling in the urothelial sensory system in the bladder. *Nat. Rev. Urol.* 13 193–204. 10.1038/nrurol.2016.13 26926246 PMC5257280

[B69] Merrill L. Vizzard M. A. (2014). Intravesical TRPV4 blockade reduces repeated variate stress-induced bladder dysfunction by increasing bladder capacity and decreasing voiding frequency in male rats. *Am. J. Physiol. Regul. Integr. Comp. Physiol.* 307 R471–R480. 10.1152/ajpregu.00008.2014 24965792 PMC4137152

[B70] Min Y. He P. Wang Q. Jin X. Song B. Li L. (2011). The effects of the c-kit blocker glivec on the contractile response of urinary bladder. *J. Surg. Res.* 171 e193–e199. 10.1016/j.jss.2011.07.048 21962730

[B71] Monaghan K. P. Johnston L. McCloskey K. D. (2012). Identification of PDGFRα positive populations of interstitial cells in human and guinea pig bladders. *J. Urol.* 188 639–647. 10.1016/j.juro.2012.03.117 22704452

[B72] Mukerji G. Yiangou Y. Grogono J. Underwood J. Agarwal S. K. Khullar V. (2006). Localization of M2 and M3 muscarinic receptors in human bladder disorders and their clinical correlations. *J. Urol.* 176 367–373. 16753445 10.1016/S0022-5347(06)00563-5

[B73] Neuhaus J. Pfeiffer F. Wolburg H. Horn L. C. Dorschner W. (2005). Alterations in connexin expression in the bladder of patients with urge symptoms. *BJU Int.* 96 670–676. 10.1111/j.1464-410X.2005.05703.x 16104929

[B74] Parajuli S. P. Soder R. P. Hristov K. L. Petkov G. V. (2012). Pharmacological activation of small conductance calcium-activated potassium channels with naphtho[1,2-d]thiazol-2-ylamine decreases guinea pig detrusor smooth muscle excitability and contractility. *J. Pharmacol. Exp. Ther.* 340 114–123. 10.1124/jpet.111.186213 22001258 PMC3251021

[B75] Preis L. Herlemann A. Adam R. M. Dietz H. G. Kappler R. Stehr M. (2015). Platelet derived growth factor has a role in pressure induced bladder smooth muscle cell hyperplasia and acts in a paracrine way. *J. Urol.* 194 1797–1805. 10.1016/j.juro.2015.05.092 26055827

[B76] Roosen A. Apostolidis A. Elneil S. Khan S. Panicker J. Brandner S. (2009a). Cadherin-11 up-regulation in overactive bladder suburothelial myofibroblasts. *J. Urol.* 182 190–195. 10.1016/j.juro.2009.02.148 19450843

[B77] Roosen A. Datta S. N. Chowdhury R. A. Patel P. M. Kalsi V. Elneil S. (2009b). Suburothelial myofibroblasts in the human overactive bladder and the effect of botulinum neurotoxin type A treatment. *Eur. Urol.* 55 1440–1448. 10.1016/j.eururo.2008.11.009 19054608

[B78] Rosenberg J. Byrtus M. Stengl M. (2016). Original Research: combined model of bladder detrusor smooth muscle and interstitial cells. *Exp. Biol. Med.* 241 1853–1864. 10.1177/1535370216655402 27328937 PMC5027948

[B79] Roskoski R. Jr. (2018). The role of small molecule platelet-derived growth factor receptor (PDGFR) inhibitors in the treatment of neoplastic disorders. *Pharmacol. Res.* 129 65–83. 10.1016/j.phrs.2018.01.021 29408302

[B80] Sancho M. Triguero D. Lafuente-Sanchis A. Garcia-Pascual A. (2017). Proliferation of interstitial cells in the cyclophosphamide-induced cystitis and the preventive effect of Imatinib. *Biomed Res. Int.* 2017:3457093. 10.1155/2017/3457093 PMC5494099 28698872

[B81] Schnegelsberg B. Sun T. T. Cain G. Bhattacharya A. Nunn P. A. Ford A. P. (2010). Overexpression of NGF in mouse urothelium leads to neuronal hyperinnervation, pelvic sensitivity, and changes in urinary bladder function. *Am. J. Physiol. Regul. Integr. Comp. Physiol.* 298 R534–R547. 10.1152/ajpregu.00367.2009 20032263 PMC2838659

[B82] Smet P. J. Jonavicius J. Marshall V. R. de Vente J. (1996). Distribution of nitric oxide synthase-immunoreactive nerves and identification of the cellular targets of nitric oxide in guinea-pig and human urinary bladder by cGMP immunohistochemistry. *Neuroscience* 71 337–348. 10.1016/0306-4522(95)00453-x 9053789

[B83] Stein P. C. Pham H. Ito T. Parsons C. L. (1996). Bladder injury model induced in rats by exposure to protamine sulfate followed by bacterial endotoxin. *J Urol.* 155 1133–1138. 8583579

[B84] Steiner C. Gevaert T. Ganzer R. De Ridder D. Neuhaus J. (2018). Comparative immunohistochemical characterization of interstitial cells in the urinary bladder of human, guinea pig and pig. *Histochem. Cell Biol.* 149 491–501. 10.1007/s00418-018-1655-z 29464320

[B85] Sui G. P. Wu C. Fry C. H. (2006). Characterization of the purinergic receptor subtype on guinea-pig suburothelial myofibroblasts. *BJU Int.* 97 1327–1331. 10.1111/j.1464-410X.2006.06200.x 16686733

[B86] Tooke K. Girard B. Vizzard M. A. (2019). Functional effects of blocking VEGF/VEGFR2 signaling in the rat urinary bladder in acute and chronic CYP-induced cystitis. *Am. J. Physiol. Renal Physiol.* 317 F43–F51. 10.1152/ajprenal.00083.2019 30995112 PMC6692721

[B87] Wiseman O. J. Fowler C. J. Landon D. N. (2003). The role of the human bladder lamina propria myofibroblast. *BJU Int.* 91 89–93. 10.1046/j.1464-410x.2003.03802.x 12614258

[B88] Yoshikawa S. Oguchi T. Funahashi Y. de Groat W. C. Yoshimura N. (2012). Glycine transporter type 2 (GlyT2) inhibitor ameliorates bladder overactivity and nociceptive behavior in rats. *Eur Urol.* 62 704–712. 10.1016/j.eururo.2012.01.044 22341128 PMC3414688

