# Supplementary figures and images for: Imatinib Mesylate Reduces Voiding Frequency in Female Mice With Acute Cyclophosphamide-Induced Cystitis
Source: Front Syst Neurosci. 2022 May 13;16:867875. doi: 10.3389/fnsys.2022.867875 (PMC9135974; doi:10.3389/fnsys.2022.867875)

## Slide 1
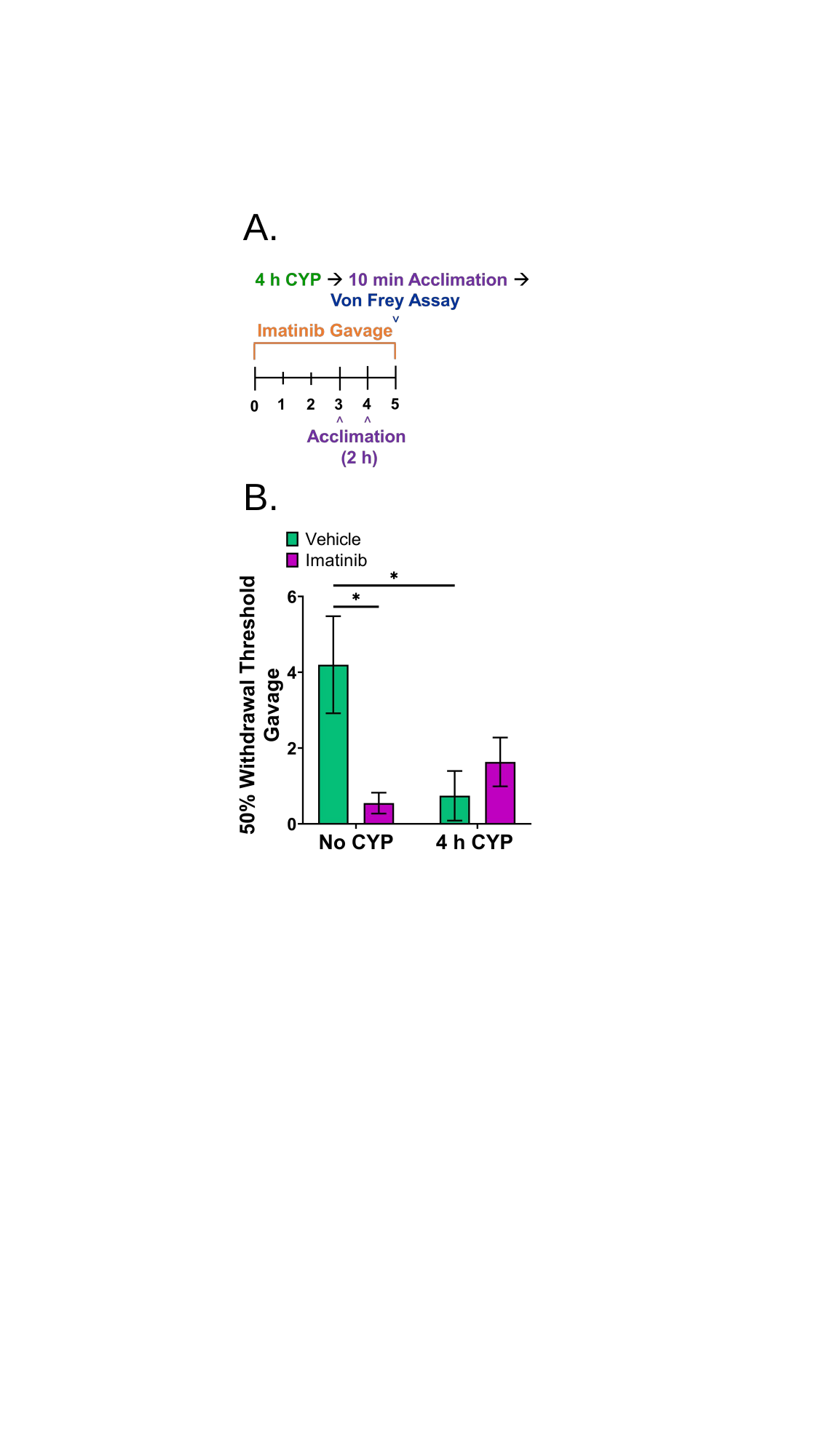

A.
B.

## Slide 2
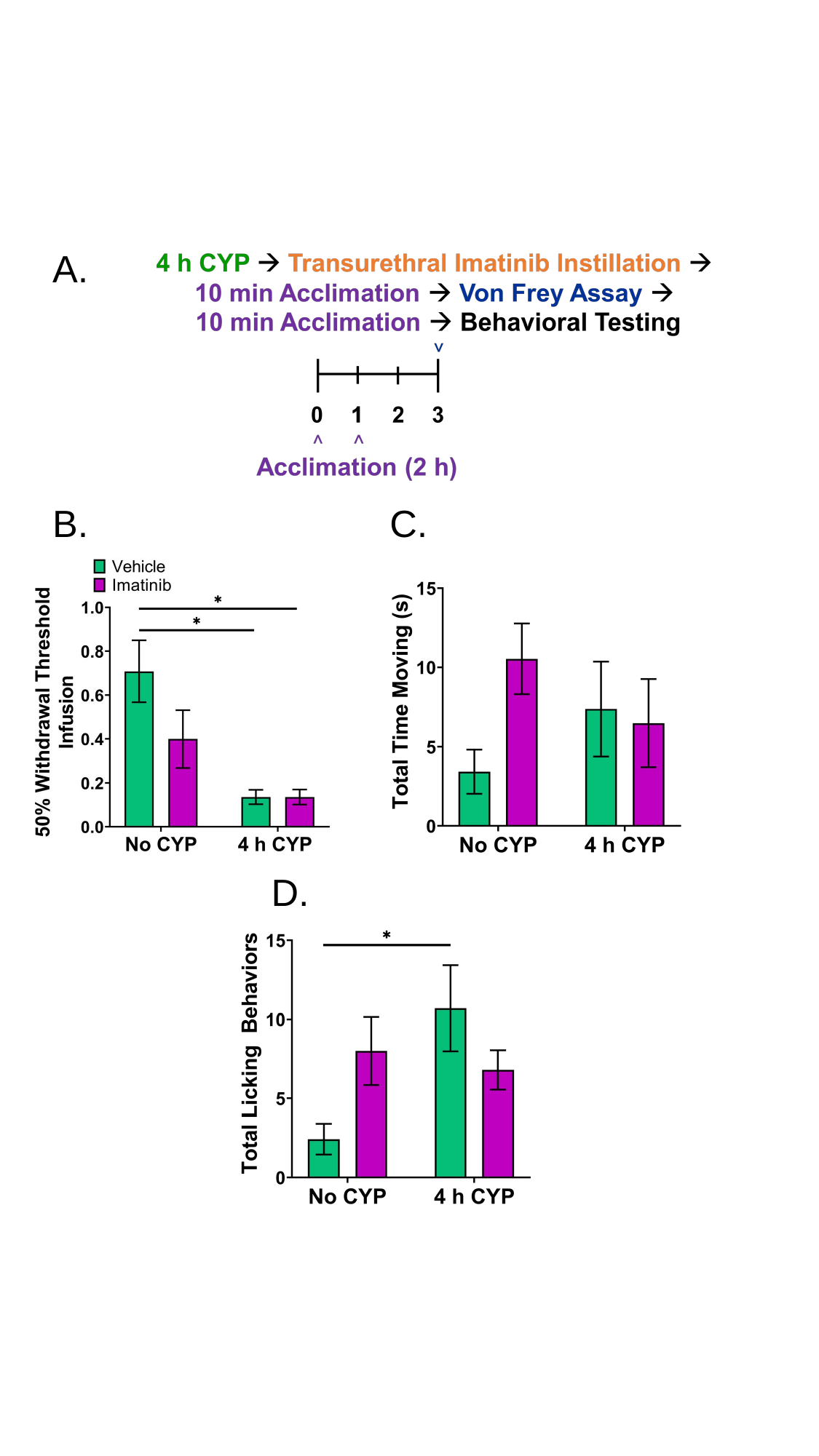

A.
D.
B.
C.

## Slide 3
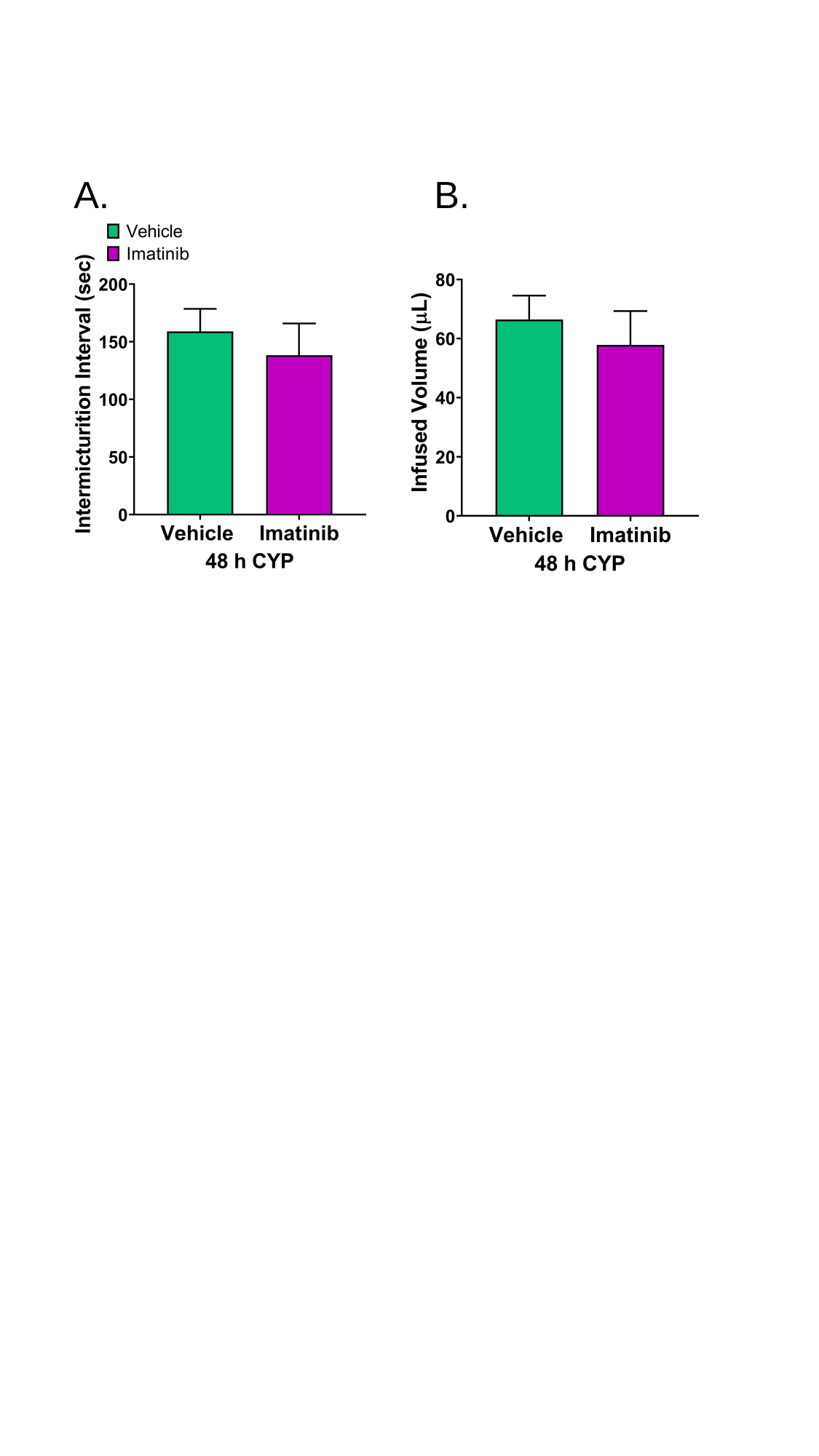

A.
B.

Supplement: Supplementary file 1 [file Presentation_1.pptx]
